# Digitally guided diode-laser cheiloplasty for correction of upper-lip asymmetry: a case report

**DOI:** 10.3389/froh.2026.1847785

**Published:** 2026-06-10

**Authors:** Ruchi Ramanujan, Tripthi P. Shetty, Rahul Bhandary, Uday Putta Simha, Nina Shenoy, Richik Chakraborty

**Affiliations:** 1 Department of Periodontology, AB Shetty Memorial Institute of Dental Sciences (ABSMIDS), Nitte (Deemed to be University), Mangalore, India; 2 Department of Oral and Maxillofacial Surgery, AB Shetty Memorial Institute of Dental Sciences (ABSMIDS), Nitte (Deemed to be University), Mangalore, India

**Keywords:** cheiloplasty, digital symmetry analysis, diode laser, ImageJ, lip asymmetry

## Abstract

**Background:**

Lip asymmetry, even subtle, can influence facial expression and self-confidence. While conventional cheiloplasty is associated with higher morbidity, diode lasers offer precise soft-tissue contouring with rapid recovery, and digital symmetry analysis enhances surgical planning.

**Methods:**

A 35-year-old woman presented with mild right-sided upper-lip asymmetry accentuated by a labially placed lateral incisor. Seeking an alternative to orthodontic treatment, she took an ImageJ-based assessment to map Cupid's-bow alignment and quantify the deviation. Using this digital guide, gentle contouring of the right upper lip was performed with a 940-nm diode laser as a conservative soft-tissue aesthetic refinement.

**Results:**

At one week, the upper-lower lip ratio improved from 1:1.73 to a more harmonious 1:1.58 with enhanced commissural symmetry. At three months, the corrected contour was maintained clinically.

**Conclusion:**

Digitally guided diode-laser contouring may provide a conservative, case-specific option for aesthetic refinement of minor lip asymmetry in selected patients.

## 1 Introduction

The smile is a universal form of communication, with the lips serving as the central, defining feature of the lower face. Lips are essential for aesthetics and function, playing a pivotal role in facial expression, speech, and oral competence (1–3). Even minor irregularities in lip contour or symmetry can disproportionately affect an individual's self-perception and psychological well-being (4). While cosmetic and functional lip deformities may result from congenital, traumatic, or iatrogenic causes, their correction still remains a significant concern in both aesthetic and reconstructive medicine.

Traditionally, surgical correction of lip deformities, such as cheiloplasty, has been performed using scalpels. But these methods can lead to complications such as significant bleeding, postoperative oedema, and an extended healing period, which may be associated with scarring (5). To address these limitations, modern dentistry has increasingly adopted laser technology for soft-tissue procedures. Lasers offer a variety of advantages over conventional surgical instruments, including a reduced need for anaesthesia, minimal pain, faster healing times, and reduced risk of infection due to their haemostatic and bactericidal properties. The haemostatic effect of lasers is a major benefit, as it creates a bloodless surgical field, allowing enhanced precision and visualisation during the procedure (6, 7). However, laser-assisted procedures also have practical and technical limitations, including higher equipment cost, limited availability, the need for operator training, and the possibility of thermal tissue injury if inappropriate parameters or technique are used (8).

Within these considerations, diode lasers have been widely investigated. Studies have demonstrated the efficacy of diode lasers in various soft-tissue procedures, from lesion excision and gingivectomy to frenectomy and, more recently, cosmetic recontouring (9). The 940 nm diode laser, in particular, has been used as a versatile tool due to its high absorption in haemoglobin and water, making it suitable for soft-tissue incisions and coagulation (6).

Digital tools such as ImageJ software Version 1.54 (National Institutes of Health, Bethesda, MD, USA) have strengthened aesthetic planning by enabling accurate measurement of symmetry, proportionality, and anatomical landmarks, allowing clinicians to map deviations more objectively and tailor interventions more precisely (10, 11).

While laser-assisted soft-tissue procedures and digital image-analysis tools have been well documented individually, and their combined use has been explored in selected aesthetic and surgical applications (9, 11), reports describing their integration specifically for reduction cheiloplasty and lip-contour refinement remain limited. In this context, ImageJ-based proportional analysis may serve as a useful adjunct for planning and documenting diode-laser-assisted soft-tissue contouring in selected cases requiring aesthetic correction.

This case report describes a 940 nm diode-laser-assisted cheiloplasty that was planned using ImageJ software for digital symmetry analysis, highlighting the role of quantitative computerised planning in guiding precise soft-tissue contouring and documenting improvements in lip proportion and commissural symmetry.

## 2 Case presentation

A 35-year-old woman presented with aesthetic concerns regarding her upper lip, noting incomplete right-sided closure. Her medical history revealed controlled hypotension managed with medication on an as-needed basis. Previous blood investigations were within normal limits, and she reported no known drug allergies.

**Figure 1:**
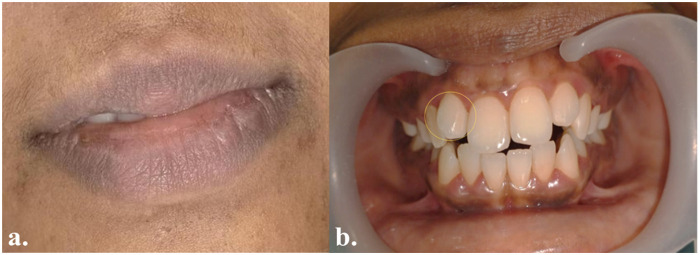
Preoperative clinical images showing: **(a)** extraoral view demonstrating lip incompetence with asymmetry of the upper-lip tubercle; **(b)** intraoral view showing a labially positioned maxillary right lateral incisor (circled) contributing to the observed upper-lip asymmetry.

Clinical examination showed mild lip incompetence and upper-lip asymmetry associated with unequal tubercular prominence. In the absence of a universally accepted clinical grading scale for lip asymmetry, the asymmetry in the present case was classified as mild, based on the presence of a visible but localised upper-lip contour discrepancy, absence of major functional impairment such as speech difficulty, drooling, or severe oral incompetence, and the feasibility of correction through conservative mucosal contouring rather than full-thickness reconstructive cheiloplasty. For clinical distinction, moderate asymmetry would have been considered to involve more evident vermilion or commissural imbalance requiring wider soft-tissue correction, while severe asymmetry would include marked tissue excess or deficiency, scar-related deformity, significant lip incompetence, or functional compromise requiring reconstructive intervention. In the present patient, the asymmetry was mainly limited to the upper-lip tubercular contour: the left side displayed a fuller, well-defined tubercle, while the right appeared less defined. A labially positioned maxillary right lateral incisor further accentuated the imbalance (Figures 1a,b).

Orthodontic correction or extraction was discussed to address the dental component, but she declined due to aesthetic, time-related, and invasive concerns. Therefore, a conservative treatment plan was formulated to improve lip harmony without altering tooth position. The patient was informed that, since the underlying dental contributor was not corrected, the planned procedure would serve as a soft-tissue aesthetic refinement rather than definitive correction of the dental component.

## 3 Treatment strategy

**Figure 2:**
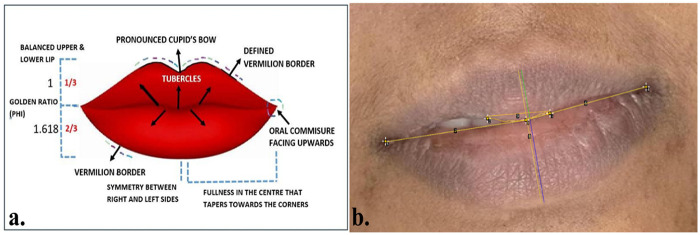
Treatment planning images showing: **(a)** schematic representation of ideal lip proportions based on the golden ratio (Φ), redrawn and modified for explanatory purposes; **(b)** digital symmetry analysis performed using ImageJ software to assess lip proportions and midline alignment.

According to Kruse-Lösler et al. reduction cheiloplasty effectively corrects persistent macrocheilia and improves lip aesthetics in patients with conditions such as Melkersson-Rosenthal syndrome (12). The golden ratio (1:1.618) has similarly been used to guide ideal lip proportions and aesthetic harmony (Figure 2a) (13).

Aligning these principles with the patient's aesthetic expectations and clinical constraints, a reduction cheiloplasty was planned using a diode laser to precisely sculpt the soft-tissue imbalance while preserving the natural vermilion contour.

The planning phase involved a golden-ratio-based assessment and individualised digital measurements aligned with the facial midline and tubercular prominence. This digital analysis helped visualise the existing symmetry and determine the extent of proportional correction required to achieve harmony with the dental and facial axes.

Since the patient declined orthodontic correction and preferred a less invasive option, a conservative protocol using a 940 nm diode laser was selected for the soft-tissue contour refinement. This approach was planned as an aesthetic camouflage procedure rather than a definitive correction of the underlying dental malposition, with the objective of improving upper-lip contour harmony and commissural balance while preserving natural lip fullness. The haemostatic property and fibre-optic delivery of 940 nm diode laser were considered advantageous for controlled soft-tissue contouring in the vascular lip region.

The patient was thoroughly counselled regarding the deformity, the planned procedure, expected outcomes, limitations, and the possible need for long-term follow-up. All details were explained verbally and in writing, and informed consent was duly obtained. The procedure was performed in accordance with institutional guidelines and relevant ethical standards.

## 4 Surgical procedure

Before surgery, routine haematological investigations were performed to ensure the patient's fitness for the procedure. As part of surgical site preparation, the perioral and lower facial regions were disinfected using 10% povidone-iodine solution, applied in outward circular strokes from the lip area to achieve optimal asepsis.

## 5 Digital symmetry and proportion analysis

Preoperative planning was done using ImageJ to measure the patient's lip asymmetry and guide precise surgical markings. A frontal photograph at rest was analysed to identify key landmarks, including the Cupid's bow peaks, philtral midpoint, and both commissures. Symmetry axes were mapped by connecting these points along the midline and commissural lines (Figure 2b).

To improve measurement consistency, all clinical photographs were obtained using a standardised frontal view with the patient seated upright in a natural head position, looking straight ahead, and maintaining the lips gently at rest. The patient was instructed to relax the facial muscles and avoid smiling, pursing, forced closure, or exaggerated lip strain before image capture. Photographs were taken using the same device, comparable lighting, similar camera angulation, and similar camera-to-patient distance.

ImageJ was then used to calculate inter-landmark distances, angles, and proportional deviations from ideal ratios. The same examiner positioned the patient, verified the resting lip posture, identified the anatomical landmarks, and performed the ImageJ measurements at baseline and follow-up. Each linear measurement was repeated three times, and the mean was used for analysis. Calibration was performed using a fixed anatomical reference within the same image wherever possible. However, because of this, a single case report based on two-dimensional clinical photography, the measurements were interpreted as proportional estimates rather than fully validated three-dimensional anthropometric measurements.

**Figure 3:**
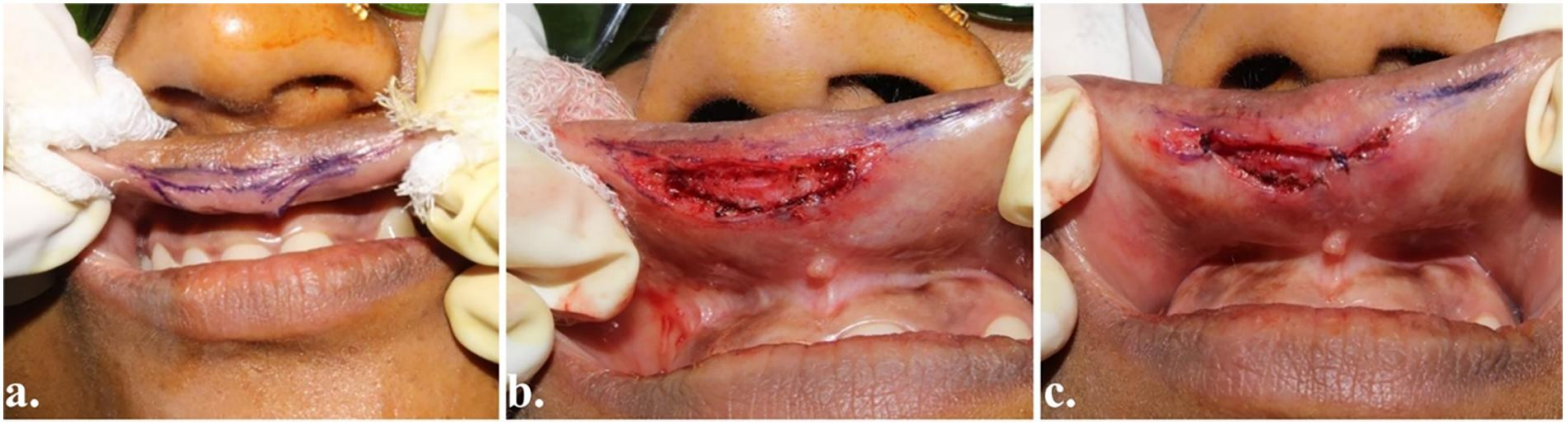
Intraoperative images showing: **(a)** preoperative marking traced according to ImageJ-based digital symmetry analysis, outlining the planned contour and commissural reference points; **(b)** diode-laser excision performed according to the digitally calculated contouring area; **(c)** placement of two interrupted sutures for partial tissue approximation to minimize postoperative contraction and support contour maintainance.

A digital outline of the planned excision zone was drawn, and its surface area was quantified using planimetry. This digitally derived design was then transferred onto the patient during surgery, allowing calibrated, data-driven markings rather than visual estimation (Figure 3a).

## 6 Clinical decision rationale

ImageJ analysis showed an upper-to-lower lip height of 130.6 px and 226.3 px, respectively, yielding a ratio of 1:1.73, slightly exceeding the commonly cited aesthetic ideal ratio of 1:1.6 (13), indicating a relatively dominant lower lip. Digital planimetry also outlined a contouring zone of roughly 240,400 px², equivalent to about 150 mm² of mucosal tissue (Table 1).

**Table 1:** Pre-op digital symmetry and proportion analysis.

*	Area	Mean	Min	Max	X	Y	Angle	Length
1	0	119	119	119	861.5	395.5	0	0
2	0	112	112	112	665.5	411.5	0	0
3	0	101	101	101	783.5	415.5	0	0
4	0	63	63	63	1,154	328	0	0
5	0	39	39	39	351.5	471.5	0	0
6	381	103.29	5.026	176.082	0	0	13.379	380.321
7	437	94.049	11.631	190.109	0	0	−172.353	435.876
8	358	144.26	0	191.576	0	0	−78.69	356.931
9	240,400	138.028	0	255	0	0	0	0
10	132	142.581	124.649	198.225	0	0	−78.518	130.614
11	227	146.688	85	178.063	0	0	−78.789	226.318

*(1 = Left cupid peak, 2 = Right cupid peak, 3 = Philtral midline, 4 = Left commissure, 5 = Right commissure, 6 = Distance of midpoint to left commissure, 7 = distance from midpoint to right commissure, 8 = Midline drawn, 9 = Planned excision area, 10 = Upper lip proportion, 11 = Lower lip proportion).

Based on these findings, a conservative diode-laser contouring approach was chosen instead of a more extensive conventional reduction approach. The goal was gentle reshaping, rather than bulk removal of the right upper-lip segment, where the labially positioned lateral incisor produced a localised contour irregularity and apparent fullness. This correction was intended to improve commissural balance and proportional harmony, while preserving lip fullness, particularly because the underlying dental contributor was not corrected.

## 7 Intraoperative marking and surgical execution

Final pre-surgical markings were made with an eosin pencil, following the digitally calibrated outline generated through ImageJ symmetry analysis. Markings were performed with the patient upright prior to local anaesthesia to avoid distortion of the natural lip curvature. Local infiltration (2% lidocaine with 1:100,000 epinephrine) was administered, and a 940 nm diode laser (Epic X™, Biolase Inc., Irvine, CA, USA) was used for incision outlining and controlled mucosal contouring with an initiated fibre tip in contact mode; the full laser parameters are summarised in Table 2. The mucosa was gently separated from the submucosa to minimise thermal spread, and the diode-laser contouring was performed according to the digitally planned outline (Figure 3b). The orbicularis oris fibres were conservatively undermined using the laser and tenotomy scissors where required. Central tubercle tissue was preserved to maintain Cupid's-bow integrity. Symmetry was repeatedly verified at rest during the procedure.

**Table 2:** Laser parameters summarized.

Parameter	Specification
Type of laser	Diode Laser (940 nm, Epic X™, Biolase Inc., Irvine, CA, USA)
Wavelength (nm)	940 nm
Emission mode	Continuous Wave
Time on/Time off	5–10 s per pass via continuous contact
Delivery system	Contact mode using initiated fiber tip
Energy distribution	Approx. 100 J/cm²
Peak power	2.5 W (incision/dissection)
Average power	1 W (marking)
Spot diameter at the focus	300 µm fiber (initiated tip)
Focus spot area	≈ 0.07 mm²
Spot diameter at the tissue	Same as focus (contact mode)
Focus-to-tissue distance	0 mm
Peak power density at spot area & tissue	∼ 35.7 W/mm²
Average power density at spot area & tissue	14.3 W/mm²
Beam divergence	None
Cooling Method	Saline-moistened gauze applied intermittently
Protective Measures	Wavelength-specific safety eyewear for patient and operator

The surgical endpoint was considered adequate when the right upper-lip contour appeared balanced with the contralateral side at rest, with preservation of vermilion continuity and without excessive thinning of the lip. Hemostasis was achieved, and closure was completed with two interrupted 4–0 sutures (Figure 3c).

## 8 Post-operative management

The patient was instructed to follow a soft diet and avoid hot, spicy, acidic and hard foods for the first 48–72 h. She was advised not to stretch, bite, massage, or manipulate the operated lip and to avoid excessive smiling, pursing, or forced lip movement during the early healing period. Gentle oral hygiene was encouraged, while direct brushing or trauma to the surgical site was avoided. Analgesics were prescribed as required, along with local application of 0.2% chlorhexidine gel for seven days. The patient was instructed to report immediately in case of bleeding, increasing pain, swelling, discharge, wound opening, altered sensation, or pigmentary change.

**Figure 4:**
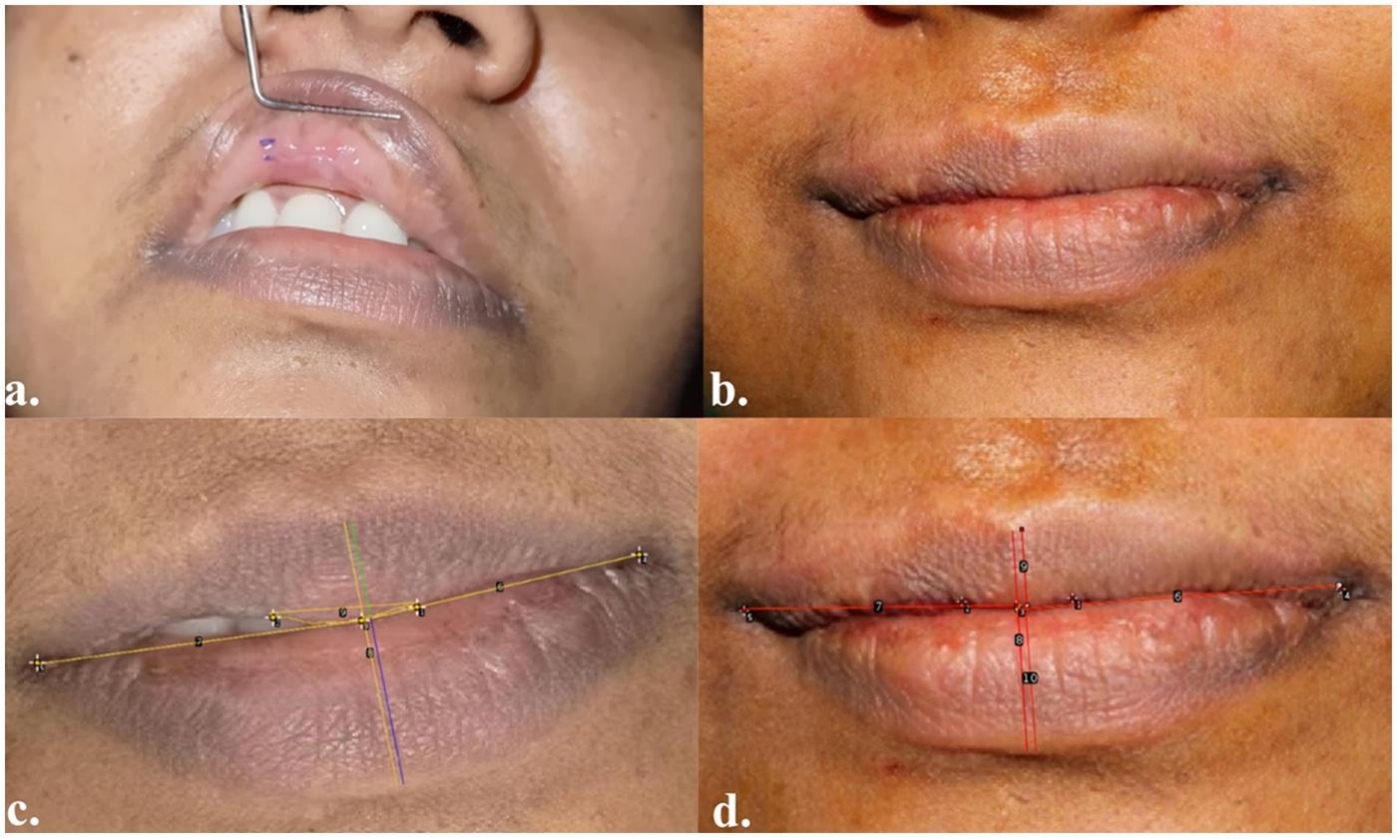
Postoperative outcomes showing: **(a)** uneventful healing at 1 week, prior to suture removal; **(b)** 3-month postoperative view demonstrating a smile line with preserved lip contour; **(c)** preoperative and **(d)** postoperative ImageJ analysis illustrating quantitative improvement in symmetry and lip proportion.

Follow-up visits were scheduled at three days for early healing assessment, one week for suture removal and epithelialization assessment, and three months for final clinical and photographic evaluation (Figures 4a,b). Healing was assessed clinically through visual inspection, gentle palpation, patient-reported symptoms, and serial clinical photography. Complete epithelialisation was considered present when the wound surface appeared continuous and intact, without ulceration, slough, bleeding on gentle manipulation, or wound dehiscence. The site was also evaluated for erythema, oedema, tenderness, fibrosis, visible scar formation, pigmentary alteration, and contour irregularity.

Digital measurements were repeated and compared using the same landmarks to evaluate proportional improvement (Table 3; Figures 4c,d). No histological or invasive healing assessment was performed, as the procedure was conservative and aesthetic in nature.

**Table 3:** Post-op digital symmetry and proportion analysis.

**	Area	Mean	Min	Max	X	Y	Angle	Length
1	0	56	56	56	857.5	395.5	0	0
2	0	90	90	90	736	411.5	0	0
3	0	69	69	69	796	415.5	0	0
4	0	45	45	45	1,154	328	0	0
5	0	25	25	25	496	471.5	0	0
6	356	87.354	0	206.873	0	0	4.201	354.954
7	303	58.659	0	255	0	0	−179.241	302.026
8	244	135.057	34.222	185.667	0	0	−86.217	242.528
9	163	138.07	0	189	0	0	−85.764	162.444
10	128	132.514	12.344	176.221	0	0	−87.320	102.820

**(1 = Left cupid peak, 2 = Right cupid peak, 3 = Philtral midline, 4 = Left commissure, 5 = Right commissure, 6 = Distance of midpoint to left commissure, 7 = distance from midpoint to right commissure, 8 = Midline drawn, 9 = Upper lip proportion, 10 = Lower lip proportion).

## 9 Results

Postoperative ImageJ analysis showed a improvement, with the right-sided commissural distance reducing from 436 px to 303 px. The upper-to-lower lip ratio improved from 1:1.73 to 1:1.58, close to the ideal golden ratio. These findings suggest that the conservative diode-laser approach achieved effective contour balancing and improved proportional aesthetic harmony in this case.

At one week, the surgical site showed complete clinical epithelialisation without wound dehiscence, visible scarring, fibrosis, or pigmentary alteration. At the three-month review, the corrected contour was maintained clinically; however, this represents a short-term outcome and cannot be interpreted as evidence of long-term stability.

No formal validated patient-reported outcome measure was used. Clinically, the patient reported minimal postoperative discomfort, no functional limitation during speech or oral competence, and satisfaction with the early aesthetic outcome at the three-month review.

## 10 Discussion

This case demonstrates the successful use of ImageJ-assisted proportional planning to guide conservative 940 nm diode-laser soft-tissue contouring in a patient with mild upper-lip asymmetry, showing uneventful healing, minimal discomfort, and no serious postoperative sequelae. The approach produced measurable short-term improvement in lip proportion and commissural symmetry; however, the result should be interpreted cautiously because the underlying dental contributor was not corrected and follow-up was limited.

LASER (Light Amplification by Stimulated Emission of Radiation) technology has transformed both the therapeutic and aesthetic aspects of dentistry since its introduction by Maiman in the 1960s, expanding into applications such as endodontic disinfection, periodontal therapy, soft-tissue surgery, frenectomy, implantology, and aesthetic soft-tissue contouring procedures (6, 7). In the present case, the diode laser was used for conservative soft-tissue contouring rather than bulk excision, aligning with the patient's preference for a less-invasive approach. ImageJ-based pre-operative symmetry analysis guided the surgical markings and helped document changes in lip proportion and commissural symmetry.

The diode laser enabled controlled incision and contouring with minimal intraoperative bleeding, allowing improved visibility in the vascular lip region (9, 14). The 940 nm wavelength's absorption in haemoglobin and melanin enabled efficient cutting and coagulation suitable for vascular lip tissue (9). Low-power, short passes minimised collateral injury, while contact mode improved control, and the laser's photobiomodulatory effect supported smooth postoperative healing (15). The laser's thermal sealing of nerve endings may have contributed to reduced postoperative discomfort, consistent with recent diode-laser studies in oral soft-tissue surgery and randomised comparisons with conventional scalpel procedures (5, 7).

Despite these advantages, diode lasers have important practical and technical limitations. Their clinical outcome depends on wavelength, power, emission mode, fibre-tip initiation, contact time, tissue pigmentation, tissue thickness, and operator experience. Compared with conventional scalpel surgery, they may increase treatment cost and require dedicated training, equipment maintenance, and laser safety precautions. Inappropriate settings, prolonged contact time, inadequate movement of the fibre tip, or insufficient cooling may increase the risk of thermal injury, tissue charring, delayed healing, or unfavourable scarring (8). In the present case, conservative settings, short passes, continuous fibre-tip movement, and intermittent saline-moistened gauze cooling were used to minimise thermal accumulation. Therefore, diode-laser surgery should be interpreted as technique-sensitive and case-dependent rather than inherently superior to conventional surgery.

The choice of a 940 nm diode laser should also be considered in relation to other wavelengths used for oral soft-tissue surgery. Laser-tissue interaction is governed primarily by wavelength-specific absorption in tissue chromophores such as water, haemoglobin, melanin, and proteins. Diode lasers in the near-infrared range, including 940 nm, are absorbed preferentially by pigmented tissues and haemoglobin, making them suitable for cutting and coagulation in vascular soft tissues. This property was advantageous in the present case because the lip is highly vascular and aesthetic precision required a clean, blood-controlled field (16).

Blue diode lasers, particularly around 445 nm, have been reported to show strong haemoglobin absorption and good haemostatic behaviour during oral soft-tissue procedures. Their interaction with superficial pigmented and vascular tissues may allow efficient cutting with favourable clinical handling; however, the evidence base for aesthetic lip contouring remains limited, and the thermal effect is still influenced by power settings, exposure duration, and tissue thickness (17).

CO₂ lasers, commonly operating around 10,600 nm, are strongly absorbed by water and therefore act more superficially on oral mucosa. This can provide precise vaporisation and favourable surface control, and some studies suggest a smaller collateral thermal damage zone with CO₂ lasers compared with diode lasers in oral mucosal excisions. However, CO₂ systems may be less accessible, more expensive, bulkier, and technique-sensitive, and their clinical effect varies according to emission mode, power density, beam delivery, and operator control (18).

Therefore, although CO₂ and blue diode lasers may offer specific advantages in selected soft-tissue applications, the 940 nm diode laser was selected in this case because of its availability, fibre-optic contact delivery, haemostatic efficiency, and suitability for conservative contouring of vascular lip tissue. The limitation remains that no direct comparison between wavelengths was performed, and therefore superiority over other laser systems cannot be claimed.

Local anaesthesia was used mainly to reduce tactile sensation from the tip, making the procedure far more comfortable than conventional scalpel techniques. Compared with electrocautery, diode lasers are documented to cause significantly less collateral thermal injury and more favourable healing, while simultaneously stimulating phagocytic and reparative activity (19). The 940 nm diode laser also facilitates mast-cell degranulation and leukocyte recruitment, accelerating wound healing and exerting a bactericidal effect that minimises secondary infection risk (20).

The hemostatic action of the diode laser, mediated through peripheral vessel coagulation and platelet activation, created a clean, bloodless surgical field, allowing superior visualisation and operative control (7).

At one week, the site showed epithelialization with no scarring, fibrosis, or pigment changes. Healing was assessed clinically through visual inspection, gentle palpation, patient-reported symptoms, and serial clinical photography; no histological or invasive healing assessment was performed. This highlights digitally guided diode-laser cheiloplasty as a conservative yet aesthetic alternative to traditional reduction surgery.

The report is limited by its single-patient design, absence of a formal validated patient-reported outcome measure, two-dimensional photographic assessment, and short follow-up period. Although the corrected contour was maintained at three months, this should be interpreted as short-term maintenance rather than evidence of long-term stability. Since scar maturation and wound remodelling may continue for several months to one year or more (21), and because lip contour is influenced by orbicularis oris activity (22) and the uncorrected dental contributor, partial relapse or contour change may occur over time. Longer follow-up at six months, one year, and beyond is therefore required to assess scar maturation, relapse, and sustained aesthetic outcome.

## 11 Conclusion

This case highlights the use of 940 nm diode-laser-assisted cheiloplasty, guided by ImageJ symmetry analysis, for conservative soft-tissue contouring in a patient with mild upper-lip asymmetry. Digital planning enabled proportionate contouring based on anatomical landmarks while preserving natural fullness. The diode laser provided haemostatic control and facilitated controlled soft-tissue handling with minimal postoperative morbidity. Together, this illustrates a contemporary, data-driven approach where objective digital analysis assists surgical planning and documentation in aesthetic lip-contouring procedures. Longer follow-up and comparative studies are required to determine the long-term stability and broader applicability of this approach.

## Data Availability

The original contributions presented in the study are included in the article/Supplementary Material, further inquiries can be directed to the corresponding author.
